# A review of the WHO malaria rapid diagnostic test product testing programme (2008–2018): performance, procurement and policy

**DOI:** 10.1186/s12936-019-3028-z

**Published:** 2019-12-02

**Authors:** Jane Cunningham, Sophie Jones, Michelle L. Gatton, John W. Barnwell, Qin Cheng, Peter L. Chiodini, Jeffrey Glenn, Sandra Incardona, Cara Kosack, Jennifer Luchavez, Didier Menard, Sina Nhem, Wellington Oyibo, Roxanne R. Rees-Channer, Iveth Gonzalez, David Bell

**Affiliations:** 1 World Health Organization (WHO), Global Malaria Programme, 20 Appia Avenue, 1211 Geneva, Switzerland; 2 Independent Consultant, Bedford Hill, Balham, London, SW12 9HR UK; 3 School of Public Health and Social Work, Queensland University of Technology (QUT), 2 George St, Brisbane, QLD Australia; 4 Malaria Branch, Division of Parasitic Diseases and Malaria, Center for Global Health, Centers for Disease Control and Prevention (CDC), Atlanta, GA 30329 USA; 5 Australian Defence Force Malaria and Infectious Disease Institute (ADFMIDI), Gallipoli Barracks Enoggera, 4051 Brisbane, Australia; 6 Department of Clinical Parasitology, Hospital for Tropical Diseases (HTD), Mortimer Market Centre, Mortimer Market, Capper St, Fitzrovia, London, UK; 7 London School of Hygiene and Tropical Medicine (LSHTM), London, UK; 8 Foundation for Innovative New Diagnostics (FIND), Campus Biotech, Building B, Level 0, Chemin des Mines 9, 1202 Geneva, Switzerland; 9 Médecins Sans Frontières (MSF), Plantage Middenlaan 14, 1018 DD Amsterdam, The Netherlands; 10 Parasitology Department of the Research Institute of Tropical Medicine (RITM), 9002 Research Dr, Alabang, Muntinlupa, The Philippines; 11 Laboratoire d’Epidémiologie Moléculaire du Paludisme, Institut Pasteur du Cambodge, Monivong Boulevard, PO 983, Phnom Penh, Cambodia; 12 Department of Medical Microbiology and Parasitology, College of Medicine, University of Lagos (UL), Private Mail Bag 12003, Lagos, Nigeria; 13 Malaria Branch, Division of Parasitic Diseases and Malaria, Center for Global Health, Centers for Disease Control and Prevention (CDC), Bldg. 23, Room 10-169, 1600 Clifton Road, Mailstop D-67, Atlanta, GA 30329 USA

**Keywords:** Malaria, *Plasmodium falciparum*, *Plasmodium vivax*, Rapid diagnostic tests, Product improvement

## Abstract

Malaria rapid diagnostic tests (RDTs) emerged in the early 1990s into largely unregulated markets, and uncertain field performance was a major concern for the acceptance of tests for malaria case management. This, combined with the need to guide procurement decisions of UN agencies and WHO Member States, led to the creation of an independent, internationally coordinated RDT evaluation programme aiming to provide comparative performance data of commercially available RDTs. Products were assessed against *Plasmodium falciparum* and *Plasmodium vivax* samples diluted to two densities, along with malaria-negative samples from healthy individuals, and from people with immunological abnormalities or non-malarial infections. Three measures were established as indicators of performance, (i) panel detection score (PDS) determined against low density panels prepared from *P. falciparum* and *P. vivax* wild-type samples, (ii) false positive rate, and (iii) invalid rate, and minimum criteria defined. Over eight rounds of the programme, 332 products were tested. Between Rounds 1 and 8, substantial improvements were seen in all performance measures. The number of products meeting all criteria increased from 26.8% (11/41) in Round 1, to 79.4% (27/34) in Round 8. While products submitted to further evaluation rounds under compulsory re-testing did not show improvement, those voluntarily resubmitted showed significant increases in *P. falciparum* (p = 0.002) and *P. vivax* PDS (p < 0.001), with more products meeting the criteria upon re-testing. Through this programme, the differentiation of products based on comparative performance, combined with policy changes has been influential in the acceptance of malaria RDTs as a case-management tool, enabling a policy of parasite-based diagnosis prior to treatment. Publication of product testing results has produced a transparent market allowing users and procurers to clearly identify appropriate products for their situation, and could form a model for introduction of other, broad-scale diagnostics.

## Background

Malaria continues to be a serious threat, responsible for approximately 435,000 deaths in 2017 [1]. Since infection with *Plasmodium* parasites causes clinical presentation indistinguishable from other fever-causing pathogens, rapid, accurate diagnosis is a crucial component of effective case management [2]. While microscopy once formed the cornerstone of parasite-based malaria diagnosis [2], most diagnosis was based on inaccurate clinical assessment. The advent of antigen-detecting point-of-care rapid diagnostic tests (RDTs) changed the landscape of diagnostic testing. RDTs are immunochromatographic lateral flow devices offering qualitative diagnosis, based on detection of parasite antigens in patient blood, such as histidine rich protein 2 (HRP2) expressed by *Plasmodium falciparum* and/or *Plasmodium* lactate dehydrogenase (pLDH) expressed by all human malaria species [3]. RDTs attracted interest since they offer accurate diagnosis while circumventing obstacles faced when using microscopy in peripheral health care settings, including cost of equipment, unstable reagents, and the need for electricity and skilled personnel (2). RDTs are relatively easy to use and provide a rapid time to result (< 30 min) [3].

The first malaria RDTs emerged in the early 1990s [4], and the World Health Organization (WHO) held its first meeting on rapid diagnostic testing in 1999 [2]. While adoption was slow, reports suggested they could be a useful tool [5]. Rapid expansion in the number of products occurred by the early 2000s. However, reports of variable field performance underscored the need to develop guidance to aid national malaria programmes on RDT procurement and implementation [6–8]. Concern regarding weak in vitro diagnostic (IVD) regulation in many endemic countries, combined with the absence of an independent evaluation process, and lack of product validation standards, led the WHO and other agencies to create an international RDT quality control programme for malaria RDTs [2], focussed around independent product testing and lot testing.

## Development of the WHO RDT evaluation programme (product testing and lot testing)

Development of a coordinated effort to quality control malaria RDTs pre-purchase (product testing) and post-purchase (lot testing) began in 2002 at the WHO Regional Office for the Western Pacific (WPRO) as a collaboration with the Special Programme for Research and Training in Tropical Diseases (TDR) and the WHO Roll Back Malaria Programme. In 2003 WPRO convened a multi-partner consultation including the Philippines Research Institute for Tropical Medicine (RITM), the Institut Pasteur du Cambodge (IPC)/Cambodian National Malaria Centre (CNM), TDR, WHO-RBM, US Centers for Disease Control and Prevention (CDC), and the Hospital for Tropical Diseases (HTD) [9]. Subsequently, standard operating procedures (SOPs) were developed, and collection of wild type *P. falciparum* and *Plasmodium vivax* samples was undertaken in 12 countries in Africa, Asia, and South America [10]. Samples were characterized by microscopy and polymerase chain reaction (PCR), followed by ELISA-based quantification of the parasite antigens HRP2, pLDH and aldolase. Only samples that contained monoinfections with *P. falciparum* and *P. vivax* and had antigen above a minimum threshold consistent with clinical infection were included [9, 11].

**Fig. 1:**
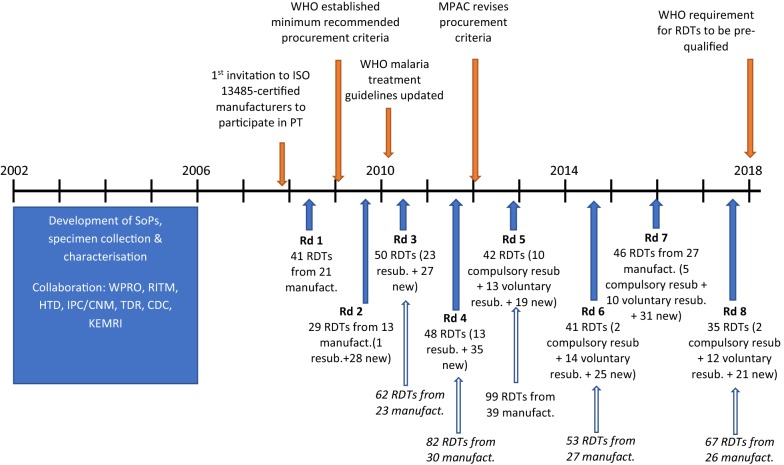
Timeline for WHO malaria RDT product testing program (PT). Number of products tested in each round, along with number of expressions of interest (italics). Solid blue arrows represent start of product testing round; open blue arrows represent response to corresponding expression of interest for rounds where expressions of interest exceeded testing capacity. *WPRO* WHO Regional Office for the Western Pacific, *RITM* Research Institute for Tropical Medicine, *HTD* Hospital for Tropical Diseases, *IPC/CNM* Institut Pasteur du Cambodge/Cambodian National Malaria Centre, *KEMRI* Kenya Medical Research Institute, *TDR* Special Programme for Research and Training in Tropical Diseases, *CDC* US Centers for Disease Control and Prevention, *MPAC* Malaria Policy Advisory Committee

After 4 years of development, specimen collection and piloting, in 2007, the WHO and the Foundation for Innovative New Diagnostics (FIND) implemented lot testing services (testing a sample of a production lot) on a limited basis at RITM and IPC/CNM. Soon after, WPRO issued recommendations that procurers only purchase products manufactured under the ISO 13485 standard, and submit a sample from each production lot, for lot-testing. However, comparative performance assessment was still needed to guide initial procurement decisions. Therefore, in 2008, the WHO invited ISO 13485-certified manufacturers to participate in the first round of ‘product testing’ to be conducted at the CDC, which assessed detection accuracy, reliability, and heat stability of commercially available RDTs, against a large panel of *P. falciparum*, *P. vivax* and negative samples, to enable WHO to develop evidence-based recommendations on product selection (Fig. 1) [12]. Following consultations in 2009, the WHO established minimum recommended procurement criteria based on these product performance evaluations and compliance with ISO 13485. A panel detection score (PDS) of ≥ 50% was recommended against the 200 parasites/μL density for *P. falciparum* and *P. vivax*, ideally higher in low-transmission settings. A false positive rate of < 10% and invalid rate of < 5% was recommended in all transmission settings. Criteria were tightened in 2012 by the WHO Malaria Policy Advisory Committee (MPAC) to a PDS of ≥ 75% against the 200 parasites/μL density for both species in all transmission settings [13].

## Overview of product testing procedures

Prior to each round of product testing, WHO issued a call for expression of interest to invite manufacturers to submit products for assessment. Manufacturers must have had a valid ISO 13485:2003 certificate to participate, and those accepted needed to submit more than 1000 RDTs from 2 lots, for each product. Evaluation was performed using cryo-preserved blood samples, with testing divided into two phases. During Phase 1, products were screened against 20 cultured *P. falciparum* parasites diluted in whole blood to 200 parasites/µL, with each sample being tested on two RDTs from each lot. A higher density of 2000 parasites/µL was also tested on one RDT from each lot. Products needed to meet a PDS of ≥ 80% against the 2000 parasites/µL density samples to proceed to Phase 2.

The Phase 2 panel comprised approximately 100 wild-type *P. falciparum* samples consisting of paired dilutions at 200, and 2000 parasites/µL, (or 5000 parasites/µL, in early panel iterations), 35 wild type *P. vivax* pairs, and 100 microscopy and PCR malaria negative samples from transmission-free populations with no recent history of exposure to malaria and half containing no known pathogens or immunological factors (clean negatives), and the other half containing pathogen and immunological factor-containing blood (dirty negatives). When wild type samples were depleted following a testing round they were replaced with new samples ensuring no statistical difference in the distribution of panel antigen concentration between rounds [10].

During evaluation, RDT results were read by two trained personnel; the first reader determined results at the minimum manufacturer stated time and the second reader as soon as possible thereafter (< 30 min). The second reader was blinded to results from the first read. Test line intensity was recorded on a scale of 0 (no band) to 4 (strong band) using standard colour charts, with intensities 1–4 classified as positive. The PDS was used as the performance measure to score products in each phase. Since Phase 1 acted as a screening step, only PDS measured in Phase 2 was used for product assessment. Results from the first read were used to determine PDS.

**Fig. 2:**
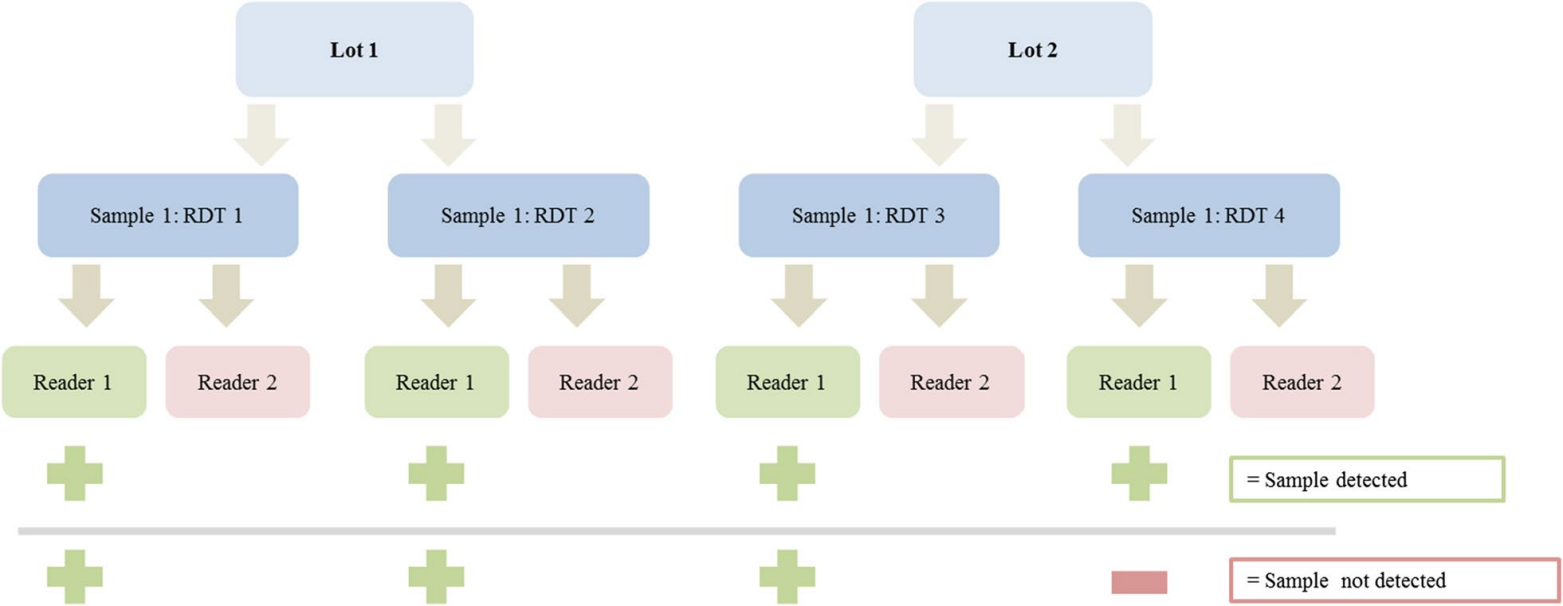
Classification of detected and undetected 200 parasite/µL samples (Adapted from the round 6 product testing report [33])

The PDS measure was developed to reflect both product sensitivity and reproducibility. It required all four tests, two from each of two manufacturing lots, against the same sample (at 200 parasites/µL) to be positive to register as “detecting” the sample, and quantifies the percentage of samples the product detected (Fig. 2). Thus it formed a more stringent measure than the more traditional measure of sensitivity.

Product false positive rate was reported, (i) overall, (ii) against each type of negative specimen, and (iii) as incorrect species detection. An invalid rate was reported for all products, with an invalid test defined as an absence of control line at the time of reading. Invalid tests were not repeated during product testing.

## Uptake of invitation to participate in WHO product testing program

The number of requests from manufacturers to submit products for testing generally increased over the eight rounds (Fig. 1). In five of the eight rounds the demand for testing exceeded the capacity of the testing laboratory and therefore each manufacturer was permitted to submit a limited number of products. In some cases manufacturers withdrew initial interest and, therefore, the final number of products tested in each round differed from the original expression of interest (Fig. 1, Table 1).

**Table 1:** Composition of products accepted to each round of testing

RDT type	Target antigen(s)	PDS species	Round (and year of testing)							
			1 (2008)	2 (2009)	3 (2010–2011)	4 (2012)	5 (2013)	6 (2014–2015)	7 (2015–2016)	8 (2016–2018)
Pf only	HRP2, Pf-LDH	Pf	16	8	15	11	9	12	19	10
Pf/pan	HRP2 or Pf,-LDH and Pan-LDH or aldolase	Pf and Pv	21	6	24	18	25	13	14	11
Pf/Pv (or Pvom)	HRP2 or Pf-LDH and Pv-LDH or Pvom-LDH	Pf and Pv	1	8	6	18	6	16	12	11
Pf, Pf and Pv	HRP2, Pf-LDH and Pv-LDH	Pf and Pv	0	0	0	0	0	0	0	1
Pf/Pv/pan	HRP2, Pv-LDH and Pan-LDH	Pf and Pv	0	2	2	0	0	0	1	0
pan only	Pan-LDH or pan-aldolase	Pf and Pv	3	4	3	1	2	0	0	2
Pv only	Pv-LDH	Pv	0	1	0	0	0	0	0	0
Total			41	29	50	48	42	41	46	35

Pf = *P. falciparum*, pan = pan specific for all malaria species, Pv = *P. vivax*, Pvom = *P. vivax, P. ovale, P. malariae*. PDS species refers to the parasite species this product was tested against, for which there is a panel detection score

In total 332 products were evaluated over the eight rounds of testing; 227 were unique [14], with the remainder (105) being resubmitted products that had been evaluated in previous rounds (Fig. 1). While some manufacturers voluntarily resubmitted products, compulsory re-testing was introduced in Round 5 to ensure products were re-evaluated at least every 5 years. This repeat assessment confirmed performance was maintained over time. Only the most recent results were included in the published WHO performance measures. Products not re-submitted to compulsory testing were removed from subsequent performance reports [10], the associated WHO information note, and the online database of results. Overall 33 products were assessed twice, 21 were evaluated three times, and five, two and one products were assessed four, five, and six times, respectively [10].

## Trends in results from WHO product testing

### Panel detection score

**Fig. 3:**
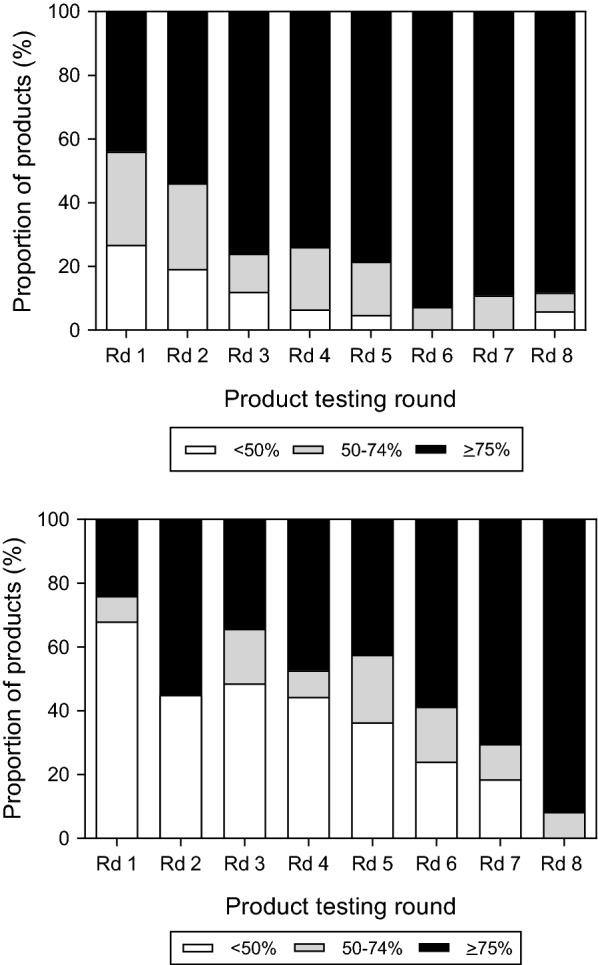
Proportion of *P. falciparum* detecting (top) and *P. vivax* detecting (bottom) products in each panel detection score category in rounds 1–8 of product testing. Bars are shaded according to the product PDS: white represents < 50%, grey: 50–74% and black, ≥ 75% (which meets the WHO recommended performance criteria). *Rd* round

Over the years of the programme, a trend of increasing PDS was observed among *P. falciparum* detecting RDTs with just under half (43.9%, 18/41) the products having PDS ≥ 75% in Round 1 compared to 88.2% (30/34) in Round 8 (Fig. 3a). For *P. vivax*, 24.0% (6/25) of Round 1 products had a PDS ≥ 75%, which increased to 91.7% (22/24) in Round 8 (Fig. 3b).

### False positivity and invalid rates

**Fig. 4:**
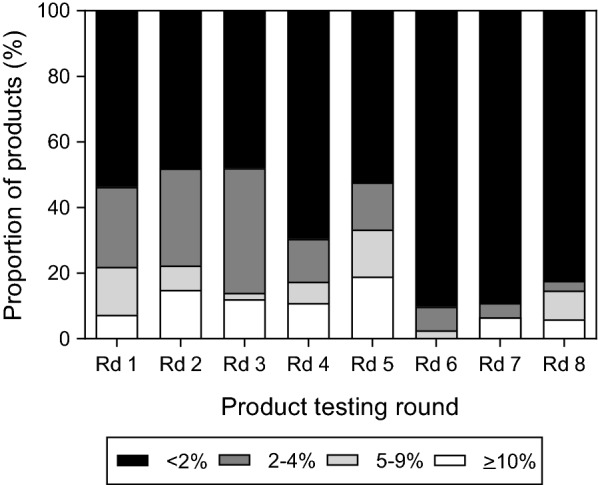
False positivity rates for products submitted to rounds 1–8. Bars are shaded according to the product false positivity rate on clean negative samples: white represents ≥ 10%, light grey: 5–9%, dark grey 2–4% and black < 2%. Only products ≥ 10% (white bars) do not meet the WHO performance criteria. *Rd* round

The false positivity rates on clean negative samples varied between rounds (Fig. 4). The proportion of products with a high false positive rate (> 10%) increased between Rounds 1–5 with 19% (8/42) of Round 5 products having > 10% false positive rate. By Round 8, this trend reversed with just 5.9% (2/34) products obtaining > 10% false positive rate. The number of products with a high invalid rate was low overall; only two products had invalid rates > 5%.

### Products meeting all WHO recommended performance criteria

**Fig. 5:**
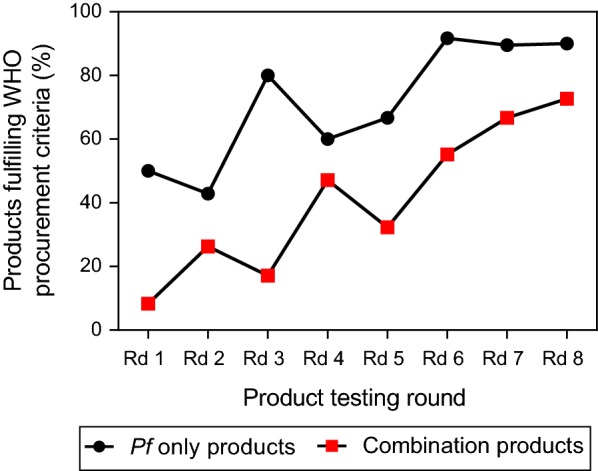
Proportion of products fulfilling WHO recommended performance criteria in each round of evaluation. Separate lines are shown for *P. falciparum* only detecting products (black circles), and combination products (red squares). One pan-only RDT assessed in Round 2, two pan-only RDTs evaluated in Round 5, two pan-only RDTs evaluated in Round 8, and one *P. vivax*-only RDT assessed in Round 2 met the WHO procurement criteria, but are not included in the figure. *Rd* round. Products are assessed against *P. falciparum* and *P. vivax* samples diluted to 200 parasites/µL

As of Round 8, 89 products have met all three performance criteria, including 36 *P. falciparum*, 26 *P. falciparum* and pan, 21 *P. falciparum* and *P. vivax*/Pvom (*vivax, malariae, ovale*), 4 pan only, one product detecting *P. falciparum* on one line with a separate line detecting *P. falciparum* and *P. vivax* together and one product detecting *P. falciparum* on one line with a separate line detecting *P. vivax* and pan.. Between Rounds 1–8, the proportion of products eligible for procurement based on performance indicators more than tripled from approximately 25% to > 80% (Fig. 5). Since combination RDTs detecting both *P. falciparum* and *P. vivax* must have a PDS meeting the WHO criteria for both species, a lower proportion of combination RDTs tend to meet the performance criteria.

### Compulsory retesting

Twenty-two, 19, 30 and 27 products were due for compulsory resubmission in Rounds 5 through 8. However, only 19 of these were actually resubmitted; 10 in Round 5, two in Round 6, five in Round 7 and two in Round 8. Results from the first and last evaluations are summarized in Table 2. Among the 19 compulsory resubmitted products, the *P. falciparum* PDS significantly decreased with a median change of 6.8% (IQR: 2.5–8.4; Wilcoxon Signed Rank Test, p = 0.006). Only eight of these 19 products detected *P. vivax*, and all except one were above the recommended PDS threshold of ≥ 75%. There was no significant change in the *P. vivax* PDS (median change = − 0.4%, IQR: − 10.0 to 5.4; Wilcoxon Signed Rank Test, p = 0.273). Overall there was a significant decrease in median false positive rate of 1.6% (IQR: 0–2.6, Wilcoxon Signed Rank Test, p = 0.033). Seventeen out of 19 products met the procurement criteria on either initial or repeat evaluation, with 12 meeting the criteria at both evaluation points.

**Table 2:** Change in panel detection score and clean negative false positivity rate for voluntarily and compulsorily resubmitted products (rounds 1–8)

Product	Catalogue number	Type of submission	First test				Last test				Change between first and last test			Met all WHO procurement criteria	
			Rd	Pf PDS (%)	Pv PDS (%)	FP (%)	Rd	Pf PDS (%)	Pv PDS (%)	FP (%)	Pf PDS (%)	Pv PDS (%)	FP (%)	1st test	Last test
Advanced Quality™ One Step Malaria Pf Test	ITP11002TC1/TC40	C	1	57.0	N/A	16.1	5	53.0	N/A	7.7	− 4.0	N/A	− 8.4	No	No
Advanced Quality™ One Step Malaria Pf Test	ITP11002TC1/TC40	V	5	53.0	N/A	7.7	7	93.0	N/A	0.4	40.0	N/A	− 7.3	No	Yes
Advanced Quality™ One Step Malaria (Pf/Pv) Tri-line Test (whole blood)	ITP11003 TC40	V	3	86.9	0.0	18.5	7	92.0	97.1	0.4	5.1	97.1	− 18.1	No	Yes
Advantage Mal Card	IR221025	C	1	62.0	100.0	4.2	5	30.0	94.3	0.4	− 32.0	− 5.7	− 3.8	No	No
Advantage P.f. Malaria Card	IR016025	C	1	97.5	N/A	0.0	5	89.0	N/A	0.0	− 8.5	N/A	0.0	Yes	Yes
Advantage Pan Malaria Card	IR013025	C	1	72.2	100.0	1.8	5	77.0	100.0	0.4	4.8	0.0	− 1.4	No	Yes
Asan Easy Test^®^ Malaria Pf/Pan Ag	AM4650-K	V	5	81.0	34.3	0.4	7	88.0	100.0	1.3	7.0	65.7	0.9	No	Yes
BIONOTE MALARIA P.f. Ag Rapid Test Kit	RG19-11	V	3	85.9	N/A	2.0	6	88.0	N/A	0.5	2.1	N/A	− 1.5	Yes	Yes
BIONOTE MALARIA P.f & Pan Ag Rapid Test Kit	RG19-08	V	3	93.9	88.6	3.0	6	83.0	68.6	0.5	− 10.9	− 20.0	− 2.5	Yes	No
BioTracer™ Malaria P.f/PAN Rapid Card	17,012	V	5	77.0	77.1	0.4	7	96.0	97.1	0.9	19.0	20.0	0.5	Yes	Yes
CareStart™ Malaria HRP2 (Pf)	RMOM-02571^b^	C	1	98.7	N/A	2.4	5	91.0	N/A	0.9	− 7.7	N/A	− 1.5	Yes	Yes
CareStart™ Malaria HRP2 (Pf)	RMOM-02571^b^	V	5	91.0	N/A	0.9	8	92.0	N/A	0.0	1.0	N/A	− 0.9	Yes	Yes
CareStart™ Malaria HRP2/pLDH Pf	RMPM-02571^e^	C	2	98.0	N/A	3.0	6	91.0	N/A	0.0	− 7.0	N/A	− 3.0	Yes	Yes
CareStart™ Malaria HRP2/pLDH Pf	RMPM-02571^e^	V	6	91.0	N/A	0.0	8	96.0	N/A	0.0	5.0	N/A	0.0	Yes	Yes
CareStart™ Malaria HRP2/pLDH (Pf/PAN) COMBO	RMRM-02571^d^	C	1	97.5	90.0	3.0	5	90.0	94.3	0.4	− 7.5	4.3	− 2.6	Yes	Yes
CareStart™ Malaria HRP2/pLDH (Pf/PAN) COMBO	RMRM-02571^d^	V	5	90.0	94.3	0.4	8	87.0	94.3	0.0	− 3.0	0.0	− 0.4	Yes	Yes
CareStart Malaria HRP2/pLDH (Pf/Pv) COMBO	RMVM-02571^a^	V	2	90.0	90.0	0.5	8	87.0	100.0	0.0	− 3.0	10.0	− 0.5	Yes	Yes
CareStart Malaria HRP2/pLDH (Pf/VOM) COMBO	RMWM-02571^c^	V	2	89.0	80.0	0.5	4	89.8	91.2	0.0	0.8	11.2	− 0.5	Yes	Yes
CareStart Malaria HRP2/pLDH (Pf/VOM) COMBO	RMWM-02571^c^	C	4	89.8	91.2	0.0	8	87.0	100.0	0.0	− 2.8	8.8	0.0	Yes	Yes
CareStart™ Malaria pLDH (PAN)	G0111	C	1	92.4	100.0	6.6	5	84.0	88.6	0.0	− 8.4	− 11.4	− 6.6	Yes	Yes
CareStart™ Malaria Pf/PAN (pLDH) Ag RDT	RMLM-05071^f^	C	3	88.9	91.4	0.5	7	73.0	0.0	1.7	− 15.9	− 91.4	− 0.5	Yes	No
CareStart™ Malaria Pf/PAN (pLDH) Ag RDT	RMLM-05071^f^	V	7	73.0	0.0	1.7	8	83.0	97.1	1.0	10.0	97.1	− 0.7	No	Yes
CareStart™ Malaria Screen RDT	RMAM-05071 ^g^	C	3	86.9	88.6	2.5	7	93.0	94.3	0.0	6.1	5.7	− 2.5	Yes	Yes
CareStart™ Malaria Pf (HRP2/pLDH) Ag Combo 3-line RDT	RMSM-XXX7X	V	7	94.0	N/A	0.4	8	82.0	N/A	0.5	− 12.0	N/A	0.1	Yes	Yes
diagnosticks—Malaria (Pf)Cassette WB	KMFC6001	V	2	59.0	N/A	7.0	5	88.0	N/A	0.9	29.0	N/A	− 6.1	No	Yes
EzDx™ Malaria Pan/Pf Rapid Test Detection kit	RK MAL 001	V	4	76.5	11.8	3.5	6	78.0	88.6	1.4	1.5	76.8	− 2.1	No	Yes
Falcivax Rapid Test for Malaria Pv/Pf (device)	503010025^j^	V	2	92.0	45.0	4.5	8	95.0	100.0	0.5	3.0	55.0	− 3.0	No	Yes
First Response^®^ Malaria Ag Combo (pLDH/HRP2)	I16FRC30	V	1	100.0	75.0	3.6	5	85.0	74.3	0.0	− 15.0	− 0.7	− 3.6	Yes	No
First Response Malaria Ag P. falciparum (HRP2) Card	I13FRC30	C	1	100.0	N/A	3.0	5	95.0	N/A	0.4	− 5.0	N/A	− 2.6	Yes	Yes
First Response^®^ Malaria Ag. P.f./P.v. Card test	PI19FRC25	V	6	85.0	71.4	0.5	8	94.0	100.0	1.0	9.0	28.6	0.5	No	Yes
FirstSign™—ParaView (Pan + Pf) Malaria Test	2101 CB-25	V	2	85.0	80.0	25.5	4	87.8	61.8	2.6	2.8	− 18.2	− 22.9	No	No
Humasis Malaria P.f/Pan Antigen Test	AMAL-7025	V	4	100.0	0.0	97.8	5	90.0	91.4	0.9	− 10.0	91.4	− 96.9	No	Yes
ICT Malaria Dual Test	ML03	V	3	78.8	60.0	0.5	7	85.0	31.4	1.3	6.2	− 28.6	0.8	No	No
ICT Malaria Pf Cassette Test	ML01	V	1	82.3	N/A	0.6	3	86.9	N/A	0.0	4.6	N/A	− 0.6	Yes	Yes
ICT Malaria Pf Cassette Test	ML01	C	3	86.9	N/A	0.0	7	94.0	N/A	1.7	7.1	N/A	1.7	Yes	Yes
ICT Malaria Combo Cassette Test	ML02	V	1	86.1	0.0	0.6	4	76.5	5.9	0.4	− 9.6	5.9	− 0.2	No	No
IMMUNOQUICK^®^ MALARIA falciparum	0502_K25	C	1	91.1	N/A	0.6	5	72.0	N/A	5.1	− 19.1	N/A	4.5	Yes	No
Malaria pf (HRP II)/(PAN-LDH) Antigen Detection Test	MFV-124R	V	1	77.2	30.0	9.5	3	95.0	0.0	5.5	17.8	− 30.0	− 4.0	No	No
Malaria pf (pLDH)/PAN-pLDH Test Device	MFV-124	V	3	2.0	5.7	0.0	5	41.0	8.6	81.3	39.0	2.9	81.3	No	No
Malaria P.f./Pan Rapid Test Cassette	IMPN-402	V	7	69.0	71.4	0.9	8	63.0	91.4	0.5	− 6.0	20.0	− 0.4	No	No
Malaria Rapid Combo/Clearview^®^ Malaria Combo	VB11	V	1	87.3	10.0	7.7	3	82.8	5.7	3.5	− 4.5	− 4.3	− 4.2	No	No
Malaria Rapid Dual/Clearview^®^ Malaria Dual Test	VB20	V	1	76.0	5.0	10.1	5	89.0	60.0	12.7	13.0	55.0	2.6	No	No
Malaria Rapid Pf/Clearview^®^Malaria Pf	VB01	V	1	68.4	N/A	0.6	5	84.0	N/A	5.1	15.6	N/A	4.5	No	Yes
Malascan™ Device—Rapid test for Malaria Pf/Pan	50,402,025	V	1	63.3	0.0	5.4	3	82.8	57.1	1.0	19.5	57.1	− 4.4	No	No
Maleriscan^®^ Malaria P.f/PAN (Pv, Pm, Po) 3 Line Antigen Test	MAT-PF/PAN-50	V	4	84.7	0.0	1.3	5	84.0	62.9	3.0	− 0.7	62.9	1.7	No	No
NanoSign Malaria Pf/Pan Ag	RMAP10	V	3	77.8	0.0	0.5	4	92.9	97.1	0.4	15.1	97.1	− 0.1	No	Yes
One Step Malaria Pf Test (Cassette)	522,352	V	2	37.0	N/A	0.5	4	94.9	N/A	1.3	57.9	N/A	0.8	No	Yes
One Step Malaria P.f Test	W37-C	V	3	60.6	N/A	0.0	7	93.0	N/A	0.0	32.4	N/A	0.0	No	Yes
One Step Malaria P.f/P.v Whole Blood Test	W056-C	V	5	87.0	28.6	2.1	7	92.0	65.7	1.3	5.0	37.1	− 0.8	No	Yes
One Step Malaria P.F/P.V Test (Cassette)	523,352	V	4	50.0	0.0	1.3	5	92.0	100.0	77.1	42.0	100.0	75.8	No	No
Onsite Pf Ag Rapid Test	R0114C	V	2	59.0	N/A	0.0	6	75.0	N/A	0.0	16.0	N/A	0	No	Yes
Onsite Malaria Pf/Pan Malaria Ag Rapid Test	R0113C	V	2	63.0	20.0	0.0	6	78.0	85.7	0.0	15.0	65.7	0	No	Yes
Onsite Malaria Pf/Pv Ag Rapid Test	R0112C	V	2	61.0	75.0	0.5	6	74.0	80.0	0.0	13.0	5.0	− 0.5	No	No
OptiMAL-IT	710,024	V	1	36.7	95.0	0.0	3	50.5	97.1	2.0	13.8	2.1	2.0	No	No
Parabank™ Device—Rapid test for Malaria Pan	50,301,025	V	1	1.3	30.0	3.0	3	17.2	62.9	0.5	15.9	32.9	− 2.5	No	No
Paracheck^®^ Pf Device—Rapid test for P. falciparum Malaria (Ver. 3)	302030025 ^k^	V	1	54.4	N/A	1.2	4	95.9	N/A	1.3	41.5	N/A	0.1	No	Yes
Paracheck^®^ Pf Device—Rapid test for P. falciparum Malaria (Ver. 3)	302030025 ^k^	C	4	95.9	N/A	1.3	8	94.0	N/A	3.4	− 1.9	N/A	2.1	Yes	Yes
Paracheck^®^ Pf Dipstick—Rapid test for P. falciparum Malaria (Ver.3)	30,302,025	V	1	74.7	N/A	7.2	4	70.4	N/A	0.9	− 4.3	N/A	− 6.3	No	No
ParaHIT^®^—f (Device)	551C104-50 ^h^	V	1	39.2	N/A	0.0	3	84.9	N/A	0.0	45.7	N/A	0.0	No	Yes
ParaHIT^®^ - f (Device)	551C104-50 ^h^	C	3	84.9	N/A	0.0	7	77.0	N/A	0.0	− 7.9	N/A	0.0	Yes	Yes
ParaHIT^®^—f (Dipstick)	551C103-50^i^	V	1	78.5	N/A	0.6	3	80.8	N/A	2.5	2.3	N/A	1.9	Yes	Yes
ParaHIT^®^—f (Dipstick)	551C103-50^i^	C	3	80.8	N/A	2.5	7	74.0	N/A	0.0	− 6.8	N/A	− 2.5	Yes	No
Parascreen™ Device—Rapid test for Malaria Pan/Pf	503030025 ^l^	V	1	50.6	25.0	1.2	8	91.0	94.3	0.5	40.4	69.3	− 0.7	No	Yes
QuickProfileTM Malaria Pf/Pv Antigen Test	71,050	V	6	78.0	25.7	0.0	7	79.0	88.6	22.9	1.0	62.9	22.9	No	No
RapiGEN BIOCREDIT Malaria Ag Pf/Pan (HRPII/pLDH)	C32RHA25^m^	V	5	77.0	77.1	4.7	7	91.0	100.0	3.9	14.0	22.9	− 0.8	Yes	Yes
SD BIOLINE Malaria Ag	05FK40	V	1	29.1	50.0	1.8	3	16.2	97.1	0.0	− 12.9	47.1	− 1.8	No	No
SD Bioline Malaria Ag P.f (HRP2/pLDH)	05FK90	V	3	87.9	N/A	2.0	8	90.0	N/A	0.0	2.1	N/A	− 2.0	Yes	Yes
SD BIOLINE Malaria Ag P.f/P.f/P.v	05FK120	V	6	85.0	91.4	0.0	8	89.0	97.1	0.0	4.0	5.7	0.0	Yes	Yes
SD BIOLINE Malaria Ag P.f/Pan	05FK60	V	1	96.2	35.0	1.2	5	94.0	91.4	0.0	− 2.2	56.4	− 1.2	No	Yes
SD Bioline Malaria Ag P.f/P.v	05FK80	C	2	96.0	95.0	3.5	6	92.0	94.3	1.9	− 4.0	− 0.7	− 1.6	Yes	Yes
SD BIOLINE Malaria Antigen	05FK50	C	1	97.5	N/A	2.4	5	95.0	N/A	0.0	− 2.5	N/A	− 2.4	Yes	Yes
Wondfo One Step Malaria Pf/Pan Whole Blood Test	W56-C	V	1	60.8	30.0	6.6	3	37.4	85.7	4.1	− 23.4	55.7	− 2.5	No	No

First and last submissions of the same type are compared

*PDS* panel detection score, *FP* false positivity, *Rd* round, resubmission type: *V* voluntary, *C* compulsory, *N/A* not applicable. The following list is the former product codes which have since been updated as detailed in the table: ^a^G0161/G0161-ET, ^b^G0141/G0141-ET, ^c^G0171/G0171-ET, ^d^G0131/G0131-ET, ^e^G0181/G0181-ET, ^f^G0121, ^g^G0231, ^h^55IC102-10, ^I^5 55IC101-10, ^j^50,300,025, ^k^30301025, ^l^50310025, ^m^C30RHA25

### Voluntary retesting

Of the 53 products voluntarily resubmitted, there was a significant improvement in mean *P. falciparum* PDS of 9.7% (95% CI 4.9–14.5%; paired t-test, p < 0.001), and a non-significant decrease in the mean false positive rate of 0.1% (95% CI − 5.9 to 5.8%; paired t-test, p = 0.98). Among the 37 *P. vivax* detecting products, significant *P. vivax* PDS improvements were observed with a mean change of 35.5% (95% CI 22.8–48.3%; paired t-test, p < 0.001). Fifteen products met the procurement criteria on initial evaluation, compared with 31 on repeat evaluation; 13 products met procurement criteria at both evaluation points.

## Reflection on impacts of product testing programme

Spawned by challenges of field studies, weak IVD regulation, and the need to expand access to high quality malaria diagnosis, the WHO Malaria RDT Product Testing Programme has over the past decade generated performance data on 332 products. Through direct feedback to manufacturers and global stakeholder dissemination and communication efforts, the Round 1 report catalysed an evolution of malaria diagnostic testing by revealing a subset of high-performing products [15]. This provided a pivotal body of evidence that supported the 2010 WHO Malaria Treatment Guidelines recommending RDTs as an acceptable alternative to microscopy. It was in fact on the basis of this data and reports of health worker competency at performing malaria RDTs [16] that WHO evidence-based policy and procurement recommendations were developed [13], which in turn informed major donor policies [10, 14, 17].

The product testing results also provided detailed information for manufacturers which sometimes resulted in changes in the instructions for use (IFU). For instance, observations from Round 1 showed the results from the second RDT read were often better than the first read at the manufacturers’ recommended reading time. This information was fed back to manufacturers, with many subsequently changing their IFU to increase the recommended reading times from 15 to 20 min.

The comprehensive testing protocol and transparent reporting of results not only facilitated product selection, but generated performance-based competition between manufacturers so as to capture a larger market share. A substantial improvement in test performance was associated with this, while prices have fallen [18, 19]. After 2010, when the WHO introduced a policy of parasite-based diagnosis by RDT or microscopy prior to treatment in all cases of suspected malaria [17], there was an upsurge in the number of manufacturers interested in participating in product testing. Allowing manufacturers to voluntarily resubmit products for testing provided a unique opportunity to observe the evolution of improved development as manufacturers strived to improve products to demonstrate a high PDS.

Beyond positive changes in RDT performance, uptake and use in practice, there is evidence that the program has influenced the RDT marketplace. Specifically, FIND conducted a manufacturer survey which showed the proportion of RDTs sold with a PDS ≥ 75% more than doubled from 23% in 2007, to 57% in 2009 and tripled by 2010 to 78%, coinciding with the release of the first and second product testing reports [20]. Driven by widespread compliance with WHO recommended performance criteria, this proportion further increased to 93% in 2014 [21]. Similarly, data gathered from major public sector RDT procurers showed a market shift towards procurement of only high performing products; while products purchased in 2009 included several with a sizable market share that did not meet performance criteria, this proportion decreased each year and since 2014 almost 100% of procured products met WHO performance criteria [19]. Furthermore, the market has consolidated around two suppliers who manufactured the highest-performing tests across several rounds of product testing [10, 18].

Between 2009 and 2019, all major public sector procurers have continuously had in place policies stating diagnostic test budgets can only be spent on RDTs that are recommended by the WHO. WHO recommendations on procurement of RDTs have evolved over the past decade being initially based on the results of product testing between 2009 and 2017, followed by a requirement for WHO prequalification for *P. falciparum*-only HRP2 RDTs in 2018 and also for RDT combination tests in 2019. An exception exists in which non-WHO prequalified RDTs, that meet performance criteria and specifically target non-HRP2 antigens, can be used in areas where *pfhrp2* deletions are prevalent as an interim measure [14, 22–24]. Several manufacturers have achieved WHO prequalification status [25]. The results of product testing, which constitutes the independent laboratory evaluation component of the prequalification process was used by the WHO PQ programme in prioritizing applications that include a product dossier, and manufacturing site inspection(s) to review the quality management system.

## Lot testing

Lot performance variation is an issue for all diagnostics. The product testing program tested RDTs from two different lots selected and supplied by manufacturers. There is no guarantee that results for the two lots submitted for evaluation are representative of every subsequent lot. Therefore, the WHO recommends both proactive and reactive post market surveillance to identify sub-standard lots prior to and/or post field deployment and continues to support needs of the global community through centralized testing at the Research Institute of Tropical Medicine, Philippines and the WHO has supported local capacity development for lot verification for malaria RDTs in Nigeria (ANDI Centre of Excellence for Malaria diagnosis, University of Lagos) and India (National Institute of Malaria Research) [26, 27].

## Conclusions

The objective of the WHO malaria RDT product testing programme was to provide independent comparative performance data to guide procurement decisions of UN agencies and WHO Member States. Through the close collaboration with FIND, CDC and several other partners, this objective has not only been repeatedly fulfilled, but the programme has influenced policy, clinical and manufacturer practice and helped shape the global market. Ultimately, it has driven improved product performance by establishing broadly accepted minimum performance criteria [22, 28, 29], making reference materials available that match that benchmark [30], and keeping the field open and regularly renewed, to encourage innovation and a competitive market. Since the programmes inception, an estimated 1.3 billion RDTs were procured in the public sector without any verified case of large-scale product/lot failure of WHO recommended products.

The RDT evaluation programme also served as a model for establishing and ensuring performance standards for RDTs detecting other diseases. To date, a leishmaniasis [31] and Ebola [32] RDT evaluation programme have been established using protocols adapted from malaria product testing. While significant gains have been made, there are still areas requiring attention to ensure effective case management, such as assessing RDT performance against *Plasmodium malariae*, *Plasmodium ovale* and *Plasmodium knowlesi*, and *P. falciparum* lacking HRP2.

## Data Availability

The datasets used and analysed during the current study are available from the corresponding author on reasonable request.
